# Influencing factors of initiation and maintenance of multidomain interventions in patients with mild cognitive impairment: a qualitative study

**DOI:** 10.3389/fneur.2025.1734487

**Published:** 2026-01-23

**Authors:** Yuxin Han, Qing Wang, Jianing Shao, Jiachen Zhang, Xiaomin Zhang, Shaoting Sheng

**Affiliations:** 1 School of Nursing, Nanjing University of Chinese Medicine, Nanjing, China; 2 School of Drum Tower Clinical Medicine, Nanjing University of Chinese Medicine, Nanjing, China; 3 School of Medicine, Jiangsu University, Zhenjiang, China; 4 Department of Nursing, Nanjing Drum Tower Hospital, Affiliated Hospital of Medical School, Nanjing University, Nanjing, China; 5 Department of Neurology, Nanjing Drum Tower Hospital, Affiliated Hospital of Medical School, Nanjing University, Nanjing, China

**Keywords:** influencing factors, mild cognitive impairment, multidomain interventions, multi-theory model, qualitative research

## Abstract

**Background:**

Mild cognitive impairment (MCI) is a critical period for the prevention of dementia, and multidomain interventions can effectively delay and improve patients’ cognitive decline. However, it remains a great challenge concerning the initiation and maintenance of interventions for MCI patients currently.

**Objective:**

To explore influencing factors of the initiation and maintenance of multidomain interventions in patients with MCI.

**Methods:**

This study was conducted with the recruitment of patients with MCI admitting to the Department of Neurology, Cognitive Center, and Cognitive Training Nursing Clinic of a Grade A Tertiary Hospital in Nanjing, as well as those identified through community screening, between September 2024 and February 2025 via purposive sampling. Data were collected via face-to-face semi-structured interviews. Meanwhile, data analysis for theme extraction in the study was performed through a deductive-inductive approach under the guidance of the multi-theory model (MTM).

**Results:**

This study extracted two themes and eleven subthemes: Behavior initiation (perceived disease threat triggering service seeking and participation; disease cognitive biases and cognitive anosognosia hindering intervention initiation; perceived benefits of interventions; cognitive expectations of rehabilitation outcomes; barriers to accessing and utilizing intervention resources; economic cost–benefit tradeoffs). Behavior maintenance (positive experiences during interventions; self-regulation during interventions; diverse external support; high time preference during interventions; impact of frailty and comorbidities).

**Conclusion:**

Multidomain interventions for patients with MCI are affected by complex and multiple factors. These factors should be considered in the clinical setting, with the formulation of targeted intervention strategies to improve patients’ participation in and adherence to multidomain interventions. Furthermore, it may contribute to the transformation of the interventions from effective to practical implementation and delay the progression of the disease.

## 1 Introduction

With the acceleration of the global aging process, the incidence of mild cognitive impairment (MCI) has increased significantly (1). The probability of developing Alzheimer’s disease without treatment is 15% (2), which brings a serious burden to society and the family. MCI represents a critical window for preventive intervention in dementia, with a cognitive reversal rate of 24% (3). Therefore, it is essential to implement measures at this stage to slow down the cognitive decline in patients. Multidomain interventions are a comprehensive intervention for the five areas most related to the risk of dementia, including cognitive training, exercise, diet, social activities, monitoring and management of vascular and metabolic risk factors (4, 5). Multidomain interventions (6–8) have been proven to delay and improve the progression of MCI; however, the effect of these interventions is highly dependent on patient participation and adherence (6). Research (7) indicated that MCI patients were unaware of the progression of their disease, and there was a notable delay between diagnosis and the first time they sought help to initiate multidomain interventions, averaging approximately 1.5–2.5 years. Meanwhile, Xu’s study (8) revealed that the participation rate of MCI patients in multidomain interventions was merely 2%, with an adherence rate of around 45%, and a follow-up rate of less than one third. It can be seen that the existing studies at home and abroad mostly focus on the validation of intervention effectiveness (9, 10) and the study of adherence (11, 12), and lack in-depth exploration of the factors affecting the initiation and maintenance of multidomain interventions, making it difficult to interpret their deep-seated reasons fully. Sharma (13) initially introduced the multi-theory model (MTM) in 2015. This model categorizes health behavior into two distinct stages: behavior initiation and behavior maintenance. It is capable of predicting and elucidating the factors influencing health behavior at each stage (14), thereby offering a fresh perspective for overcoming the challenges associated with initiating and maintaining multidomain interventions. On the one hand, the MTM aligns with the core research questions of our study. On the other hand, this theory features a concise and unified theoretical framework, serving as a sound and implementable tool (15). Developed based on the Health Belief Model, Theory of Planned Behavior, Social Cognitive Theory, and other relevant theories, the MTM has been successfully applied to the intervention of diseases such as diabetes (16), as well as the explanation of health behavior changes, including physical exercise and smoking cessation (17–19). The conceptual underpinnings and practical applications of this theory have become relatively mature, and a review study has corroborated its applicability in the field of chronic diseases (14). Based on this, this study employs MTM as the analytical framework and adopts qualitative research methods to delve deeply into the behavioral drivers and barriers of multidomain interventions in patients with MCI. This exploration aims to provide a foundation for the further development of personalized, multidimensional intervention implementation strategies, thereby facilitating the effective translation of multidomain interventions from evidence-based practice to real-world application.

## 2 Methods

### 2.1 Design

With a qualitative research design, this study conducted semi-structured interviews with MCI patients. The result presentation in this study follows the Qualitative Research Report Standard (20).

### 2.2 Participants

Via objective sampling and maximum differentiated sampling, this study recruited MCI patients who visited the Neurology Department, Cognitive Center, Cognitive Training Nursing Clinic of a Grade A tertiary hospital in Nanjing, or underwent community screening, from September 2024 to February 2025. Participants represented different engagement stages. Inclusion criteria: (1) Patients with MCI confirmed according to the diagnostic criteria outlined by the International MCI Working Group (21) and the 2018 Chinese Guidelines for the Diagnosis and Treatment of Dementia and Cognitive Disorders (Section 5): Diagnosis and Treatment of MCI (22): ① Patients subjectively reported memory decline, or identified cognitive impairment identified by experienced clinicians; ② Objective evidence of impairment in one or more cognitive domains on cognitive tests based on the Montreal Cognitive Assessment Scale (MoCA; Chinese version) (23): illiterate individuals with a MoCA score ≤ 14; individuals with 1–6 years of education with a MoCA score ≤ 20; individuals with ≥7 years of education with a MoCA score ≤ 25; ③ Possible impairment in complex instrumental activities of daily living, yet with independent daily living abilities maintained; (2) Patients aged ≥60 years and <80 years; (3) Patients with written informed consent provided. Exclusion criteria: (1) Patients diagnosed with dementia or having mental disorders; (2) Patients with other severe diseases, such as heart failure or malignant tumors; (3) Individuals with language communication barriers.

### 2.3 Data collection

The research team consisted of six researchers from Nanjing University of Chinese Medicine and Nanjing Drum Tower Hospital. All members of this team have undergone training in qualitative research methods, are well-acquainted with topics, and possess experience in interviewing MCI patients. With the implementation of an extensive literature review, the team initially drafted an interview outline based on the research objectives utilizing the MTM (13) as the theoretical framework. Subsequently, a pre-interview was carried out with one patient to refine and finalize the interview outline. The interview outline consisted of five questions described as below: ① Did you experience memory decline? Please describe your feelings and how these changes affect you. ② For patients with participation experience: Could you elaborate on your specific involvement in multidomain interventions? How did you learn about them? What factors motivated or hindered your participation? For patients without participation experience: Would you participate in multidomain interventions if you got the opportunity? What factors influence your decision-making? ③ What challenges have you encountered during your participation in multidomain interventions? Could you share your difficulties and how you addressed them? ④ Are you still actively participating in multidomain interventions? What motivates you to continue or discontinue your participation? What do you consider to be the most beneficial aspect of the intervention? ⑤ What are your expectations and requirements regarding the content and format of multidomain interventions? What kind of support and assistance do you seek for? The interview guide is detailed in the Supplementary File S1, including in-depth questions in specific fields.

### 2.4 Data analysis

Within 24 h of the interview, the recording was transcribed sentence by sentence, followed by content annotation in real-time based on the interview context. All materials were imported into the MAXQDA 2022 software for data management, with the compilation of a memorandum at the time. Rather than as a rigid coding scheme imposed on the data, MTM was used as a sensitizing framework to inform initial analytic attention. Data were independently coded, organized, and analyzed simultaneously by two researchers (YH and JS) using a combined deductive and inductive coding analysis (24, 25). The specific steps are as follows: ① A deductive approach based on the MTM and prior literature was used to outline the analytical dimensions to guide tentatively, but not constrain, subsequent analysis. The interview data were initially classified based on the two stages and six constructs in the MTM framework, but not entirely using theoretical expressions. ② The open coding of raw interview data was conducted by employing an inductive coding, uncovering new themes not covered by the MTM. Similar codes were grouped into corresponding categories for further comparison and integration, followed by matching with the predefined coding categories. Meanwhile, new coding categories would be added, following validation through group discussion, in cases where subthemes could not be matched to predefined deductive categories. ③ Based on the preliminarily extracted themes and subthemes, the original text would be re-accessed for repeated comparison and in-depth analysis of the underlying logical relationships between themes, subthemes, and textual materials. Multiple rounds of deductive-inductive cyclical validation would be conducted simultaneously. Besides internal discussions within the research team, expert (QW) assistance would be sought to ultimately reach a consensus in the event of disagreement, thereby establishing the final influencing factors. After organizing the codes into themes and subthemes, interview participants were to verify our coding results through face-to-face interviews and telephone follow-ups, which aimed to allow patients to confirm that our descriptions and theme summaries did not differ from their original intentions. Concurrently, to enhance the reliability of the findings, research team members (XZ and SS) were invited to review the coding outcomes. Five researchers (YH, JZ, QW, XZ, and SS) jointly identified the theme and subtheme through discussion. Interviews and preliminary coding proceeded concurrently, with regular discussions held by the team to assess and verify the emergence of new themes and meanings relevant to the research objectives. The structure of extracted themes, subthemes, and representative statements was stabilized by the 16th participant. Additional interview data were considered to provide supplementary insights to existing themes only, indicating thematic saturation. Given the heterogeneity of participant characteristics, additional participants would be continuously recruited to test whether additional interviews could uncover new insights within the analytical context, particularly regarding differences in intervention experiences. Noticeably, no new perspectives emerged at these analytical levels by the 19th interview, leading to a preliminary judgment of the saturation of the themes and meanings. To ensure reliability, one additional participant was recruited by the research team for a validation interview. According to the coding analysis of the 20th participant’s data, all perspectives could be categorized within the existing thematic framework. Following group discussion, there were no other new themes or reinterpretations of existing themes, confirming thematic saturation of the core analytical dimensions (26).

### 2.5 Rigor

To ensure the credibility of qualitative research, this study implemented strategies based on Ayelet Kuper and Lorelei Lingard’s evaluation criteria for qualitative studies (27), including: (1) focusing on the research question—namely, the challenges in initiating and sustaining multidomain interventions; (2) thorough preparatory work, including systematic literature reviews on MCI, multidomain interventions, and adherence to establish interview outlines and content; (3) detailed descriptions of the study background and design, inclusion/exclusion criteria for eligible participants, data collection, and analysis to facilitate replication; and (4) audit trails to ensure the reliability and completeness of data analysis.

### 2.6 Ethical considerations

Prior to the study, an official approval was given by the Ethics Committee of Nanjing Drum Tower Hospital (Approval No. 2024-757-01).

## 3 Results

Each interview lasted 35–60 min. Finally, this study enrolled 20 participants with MCI, including 11 females and 9 males. Participants with and without intervention experience offered different perspectives. Specifically, experience-free patients primarily described their anticipated barriers to and willingness to participate in the initial stages of multidomain interventions, while patients with prior experience provided specific insights into both facilitating factors and challenges during both intervention initiation and maintenance. Detailed participant information is presented in Table 1.

**Table 1:** General information of patients with MCI (*n* = 20).

Participants	Age	Sex	Marital status	Payment type	Region	Education	Intervention state	MMSE	MoCA
P1	69	Female	Married	UEBMI	City	Junior college	A	30	24
P2	72	Male	Married	UEBMI	City	Junior high school	B	30	25
P3	64	Male	Married	UEBMI	City	College	D	28	25
P4	66	Female	Married	NRCMS	Rural	Senior high school	D	29	21
P5	67	Male	Married	UEBMI	City	Senior high school	C	27	24
P6	71	Female	Married	UEBMI	City	Master	A	26	24
P7	76	Male	Married	UEBMI	City	Junior college	A	27	24
P8	60	Male	Married	NRCMS	Rural	Primary school	B	27	20
P9	70	Male	Married	UEBMI	City	College	C	24	16
P10	69	Female	Married	UEBMI	City	Secondary vocational school	D	27	21
P11	74	Male	Married	UEBMI	City	College	A	24	19
P12	65	Female	Married	UEBMI	City	College	B	28	24
P13	77	Female	Married	UEBMI	City	Secondary vocational school	A	28	21
P14	70	Female	Married	URRBMI	Rural	Senior high school	B	27	21
P15	78	Female	Widowed	UEBMI	City	Junior high school	A	27	21
P16	74	Female	Married	UEBMI	City	Junior high school	D	28	17
P17	69	Male	Married	UEBMI	City	Senior high school	A	27	23
P18	71	Male	Married	UEBMI	City	Junior high school	A	27	19
P19	74	Female	Married	UEBMI	City	Junior college	C	28	22
P20	78	Female	Married	UEBMI	City	College	A	26	20

MMSE refers to the Mini-Mental State Examination (MMSE), and MoCA refers to the Montreal Cognitive Assessment (MoCA). NRCMS (The New Rural Cooperative Medical Insurance): The NRCMS is a new basic health social security system in China, started in 2003, that combines insurance and social assistance, and targets all farmers and rural residents. URRBMI: Urban and Rural Residents’ Medical Insurance. UEBMI: Urban Employee Basic Medical Insurance. A: Maintain Intervention. B: Intervention not initiated. C: Intervention interruption. D: Abandon after a period of time following initiation.

This study extracted two themes and eleven subthemes: The first theme was behavior initiation, consisting of 6 subthemes involving perceived disease threat triggering service seeking and participation; disease cognitive biases and cognitive anosognosia hindering intervention initiation; perceived benefits of interventions; cognitive expectations of rehabilitation outcomes; barriers to accessing and utilizing intervention resources; economic cost–benefit tradeoffs. The second theme was behavior maintenance, with 5 subthemes of positive experiences during interventions; self-regulation during interventions; diverse external support; high time preference during interventions; impact of frailty and comorbidities. The themes, subthemes, and corresponding MTM constructs are shown in Table 2.

**Table 2:** Overview of themes, subthemes, and corresponding Multi-Theory Model (MTM) constructs.

Theme	MTM construct		Subtheme
Behavior initiation	Participatory dialogue	Disadvantages	Perceived disease threat triggering service seeking and participation
			Disease cognitive biases and cognitive anosognosia hindering intervention initiation
		Advantages	Perceived benefits of interventions
	Behavioral confidence		Cognitive expectations of rehabilitation outcomes
	Changes in physical environment		Barriers to accessing and utilizing intervention resources
			Economic cost–benefit tradeoffs
Behavior maintenance	Emotional transformation		Positive experiences during interventions
	Practice for change		Self-regulation during interventions
	Changes in social environment		Diverse external support
	(Subtheme not within the constructs)		High time preference during interventions
	(Subtheme not within the constructs)		Impact of frailty and comorbidities

### 3.1 Theme 1: behavior initiation

#### 3.1.1 Subtheme 1: perceived disease threat triggering service seeking and participation

Symptoms such as memory decline and reduced learning ability were perceived by a significant proportion of MCI patients. These changes, distinct from normal aging, created a perceived health threat, triggering patients’ anxiety about potential cognitive decline. This concern motivated the affected individuals to seek cognitive health services and actively participated in multidomain interventions.

The patients described their strong perception of memory problems.

*This year, I’ve been finding it incredibly hard to remember things. I forget what I just said the moment I turn away. This is just too bad, although I know memory fades with age. I’m afraid I’ll end up with dementia if this keeps up. I heard they offer screening and training here, so I came right away*. (P1)

For others, feedback from family and concerns about their future cognitive function exacerbated their anxiety.

*The kids all say I’m slow on the uptake, but honestly, I’m even more anxious inside. I’d gladly come to practice every single day, even if it’s just to maintain my current level.* (P7)

In addition to concerns about declining memory, people sometimes feel threatened by illness because their important social and physical activities are disrupted.

*I’ve loved dancing since I was young and was a key member of the Square dancing. Lately, I’ve noticed that learning new moves does not come as easily as it used to—I keep messing up and often cannot keep up. It’s been a real blow to me that I did not participate in the Square dancing performance this time…* (P17)

#### 3.1.2 Subtheme 2: disease cognitive biases and cognitive anosognosia hindering intervention initiation

Individuals with MCI often exhibited mild impairments across multiple cognitive domains (e.g., memory, language, and attention), despite variation in their level of awareness regarding such cognitive alterations among patients. Some patients interpreted early cognitive symptoms as benign or age-related, while others exhibited more severe self-perceived impairment, which might be indicative of underlying neurological dysfunction. Moreover, these individuals often hesitated or even refused when confronted with healthcare professionals’ intervention recommendations, which further hindered treatment initiation.

In terms of disease cognitive biases, some patients tended to downplay memory decline, attributing forgetfulness to normal aging, fatigue, or other situational factors.

*I do not think my memory is that bad. It’s normal to forget things as you get older, which has no significant impact on my daily work or training. I do not need it.* (P2)

*I do not think it’s a big deal……the kid’s making a mountain out of a molehill. I sometimes cannot remember things, even though they are right on the tip of my tongue. Lots of us seniors go through that.* (P8)

As for cognitive anosognosia, some patients had objective evidence of cognitive decline after assessment, yet denied having cognitive impairment.

*I’m not particularly worried myself. I think I’m perfectly normal. There are people around me with poor memory too; some forget things even more than I do. But they are also living perfectly well.* (P14)

Concerning the coexistence of disease cognitive biases and cognitive anosognosia, there was a group of patients whose narratives indicated the coexistence of both between these two extremes. These individuals acknowledged the results of objective assessments but reinterpreted them in ways that preserved a sense of normalcy, suggesting partial or fluctuating cognitive impairment.

*I underwent community screening and was advised to have cognitive impairment, with some interventions advised. But I feel perfectly fine, it’s just that I have too many household responsibilities. My train of thought keeps getting interrupted, so I forget things.* (P12)

#### 3.1.3 Subtheme 3: perceived benefits of interventions

Perceived benefits of interventions were the subjective recognition of MCI patients in the possible positive effects of participating in multidomain interventions by contacting information related to multidomain interventions or through other people’s experiences. As an internal driving force of patients’ actions, this perception of benefits would promote the Initiation of intervention.

Some patients reported observing positive changes in those who had participated in the intervention, changes that made them feel the benefits of the intervention. For example, one patient noted:

*Mr. Zhang from our neighborhood joined in, and now he seems more willing to talk and appears more alert.* (P1)

Other patients were exposed to information about multidomain interventions through medical staff and their families, and they recalled:

*The doctor explained it in great detail, saying that sticking with this approach (multidomain interventions) could potentially reverse my current condition. I thought about it, if it could just slow down my memory loss, even if only a little, I’d consider it worthwhile.* (P7)

*My daughter has gathered a lot of information for me, but I’m having trouble focusing right now. If it can truly be helpful, it would mean the world to me.* (P6)

*At first, I hesitated. Later, I heard from the nurse that many people will improve in* var*ying degrees after participating. I am willing to try, which is better than sitting and worrying.* (P5)

#### 3.1.4 Subtheme 4: cognitive expectations of rehabilitation outcomes

According to the definition of the expectation of cognitive rehabilitation outcomes, it indicated the confidence of MCI patients in delaying the decline or improving their cognitive function, especially the perception that a specific behavior (accepting multidomain interventions) would benefit the maintenance or improvement of their cognitive function.

Supported by the positive outcome expectations, patients would believe that participating in multidomain interventions would result in valuable changes, which in turn promoted their active participation and efforts.

*I have practiced in community-based activities several times. I feel very focused when I participate in the training. I can feel that I am ‘brain’ moving. I believe this intervention is beneficial for improving my memory.* (P13)

*I feel that my memory will not get worse after training slowly. I have confidence in myself and believe that I can recover better than now.* (P15)

*I attach great importance to the health of my brain. I have known people with the same symptoms as me. Their memory has improved after the intervention. I am confident that I can.* (P11)

On the contrary, patients would remain skeptical of multidomain interventions and hesitate to invest time and energy, thus hindering participation when they had negative or vague outcome expectations.

*At my age, how can I possibly manage everything perfectly? It is enough if I can eat my meals, sleep soundly, and keep my legs moving freely. I do not demand more, nor do I expect to regain the sharp memory I once had.* (P2)

*Whether my memory improves or not, it’s just so-so. Even if I try to boost it, can I really get my 20-year-old brain back? I often joke that as long as I can get by day to day and recognize my family members, that’s good enough.* (P14)

#### 3.1.5 Subtheme 5: barriers to accessing and utilizing intervention resources

In general, MCI patients’ participation in interventions may be supported by the accessibility, availability, affordability, and appropriateness of multidomain intervention resources in the physical environment. However, a significant proportion of MCI patients reported difficulty in getting access to intervention-related information and resources.

Moreover, patients were unable to receive adequate non-pharmacological interventions for cognitive impairment at primary healthcare facilities.

One of the patients said: *I’ve heard that memory can be improved through intervention, but the doctors at our local community hospital said they do not offer that service here and suggested I go to a major hospital in the city.* (P16)

Their behavioral initiation was severely hindered by a series of issues, such as transportation challenges, limited accessibility of intervention resources, high cost of certain online resources, or home-based training equipment, etc.

*The biggest challenge for me is the distance. At present, there is no specialized hospital for training near my home, so I have to travel to the city center. With all the transfers I have to make on buses and subways, I just do not feel like doing it anymore.* (P10)

*The doctor only prescribed medicine when I visited the clinic, without any description of other intervention methods, and I did not see any publicity.* (P15)

*I saw that there are devices and software that can be trained at home on the Internet. Relevant products have different prices, their introduction is not detailed, and I do not know whether they are effective or not.* (P19)

#### 3.1.6 Subtheme 6: economic cost–benefit tradeoffs

Given the extended durations and delayed outcomes of multidomain interventions, most MCI patients, prior to intervention, subjectively weighed the financial costs of engaging in the intervention against the anticipated tangible benefits. This cost–benefit analysis would produce a direct impact on their decision-making. Patients would decline participation, deeming it “not worth it,” when they perceived the financial burden of multidomain interventions as disproportionately high relative to the potential value gained.

Two of the patients explicitly stated that cost was a key factor in their willingness to participate.

*Is it free to participate in this multidomain intervention project? I’ll participate if it’s free, otherwise I will not.* (P4)

*I calculated the account and went to the city for training. The round-trip transportation plus the cost each time would be quite a lot in a month. I should seriously consider it.* (P10)

Even those patients who indicated a willingness to bear some of the economic costs remained concerned about the cost-effectiveness of multidomain interventions.

As one patient explained: *I am willing to participate in the training. It is OK to spend money. We all have medical insurance and can be reimbursed. But I have a very practical consideration: can this medicine-free intervention work?* (P9).

Some patients questioned whether paid multidomain interventions offer sufficient added value beyond familiar low-cost activities.

One patient stated: *If the memory intervention program is free, I might give it a try. Honestly, playing cards and dancing at home can also exercise my brain. Sometimes I feel it’s unnecessary to spend money on this.* (P17)

### 3.2 Theme 2: behavior maintenance

All findings related to behavior maintenance in our study were from participants with practical intervention experience, which were embodiments of real-world experiences, rather than hypothetical expectations.

#### 3.2.1 Subtheme 1: positive experiences during interventions

Positive experiences with multidomain interventions were important for developing high expectations of patients for outcomes, fulfilling their need for autonomy, competence, and belonging in disease management. With the subsequent creation of a positive feedback loop, it could further strengthen patient identification with and acceptance of the intervention program, reducing resistance and fatigue during the process. Such experiences served as a crucial intrinsic motivator for sustaining patient engagement in multidomain interventions. These experiences extend beyond mere pleasure to encompass psychological restructuring, enhanced cognitive and task-related abilities, and positive emotional reinforcement from healthcare providers. This process can reflect the emotional transformation of patients during the maintenance stage of the intervention, which is one of the main constructs of the maintenance stage of the MTM theory.

As for potential shifts in self-perception following the intervention, some participants described a psychological transformation from a passive “patient identity” to a more proactive stance.

*I noticed a changed mindset in myself after participating in the intervention. I used to treat myself as a patient. Now I feel more confident with more practice, and feel that my brain can be ‘rescued’. I look forward to doing some new challenges every day, and my mentality has also become optimistic.* (P20)

Some participants emphasized the positive emotional atmosphere within the interventional environment. Their willingness to participate would be enhanced when feeling emotionally comfortable and accepted during the training.

*I feel different here with you guys. Training here puts me in a great mood.* (P6)

One participant reported that the enjoyment and sense of accomplishment derived from the task played a positive role. Activities perceived as fun, easy to operate, and meaningful could enhance participants’ sense of competence while reducing their fatigue and perceived effort.

*Among them, I like the handmade tasks, such as the handmade building blocks and pasted paintings, which are very interesting and effortless, and I have a sense of achievement.* (P7)

In addition, trust in healthcare providers further enhanced participants’ positive experiences. Usually, participants reported feeling more pleased and engaged when intervention tasks aligned with their interests and were delivered by trusted healthcare providers.

*I feel I’ve made progress. I trust you all (the medical staff), and the tasks you have given me include things that interest me. It’s been quite enjoyable to train this way.* (P11)

*Every time I finish the training task, the nurses will sincerely praise me, which makes me feel very confident and can urge me at home. I am particularly looking forward to your task at home.* (P13)

#### 3.2.2 Subtheme 2: self-regulation during interventions

A considerable number of MCI patients actively adjusted and managed their cognition, emotion, and behavior during multidomain interventions to maintain the continuity of intervention. This adjustment included self-motivation at the cognitive level, self-counseling at the emotional level, and strategy adjustment at the behavioral level. Self-regulation maps the practice for change construct in the MTM, combining continuous correction to remove ineffective strategies and resolve obstacles. Through self-regulation, patients could better cope with challenges in the process, resulting in reduced interference of negative factors on the behavior of multidomain interventions participation, thereby providing internal support for maintaining long-term training.

Cognitive self-motivation was particularly evident when patients engaged in tasks using personal strategies.

*I began to consciously play some training games with my wife, making ‘bets’ with playing cards. Whenever I had more cards left than her, I knew my odds of winning were better than hers. I was particularly motivated at that time.* (P6)

Emotional self-counseling served as another crucial coping strategy to manage setbacks. Patients described their re-framing strategy of their emotional responses to challenges encountered during the intervention.

*Some training tasks always trip me up, but I know I cannot be in a hurry. I need to think about some strategies for passing the game. I think this is also part of the training. Follow the rules and skills I find.* (P7)

*I take my home training pretty seriously and do not need anyone to supervise me. I feel down if I ever fall behind on my cognitive training scores or fail to meet my exercise goals. In those cases, I choose to take a break and rest up before getting back to it.* (P11)

Another form of self-regulation observed in our study was an active seeking for feedback, where patients proactively sought guidance when encountering difficulties. This proactive approach indicated that patients manage their engagement by addressing obstacles early on, as described by this patient.

*Some memory games in training are difficult for me after upgrading. I take the initiative to approach the nurse for feedback and request her to adjust the training tasks accordingly. I also seek their assistance in explaining if I find something difficult to understand.* (P18)

#### 3.2.3 Subtheme 3: diverse external support

During the participation in multidomain interventions among these MCI patients, multiple external support such as family support, peer assistance and professional supervision provides a solid external guarantee system for their continuous participation in interventions. Such type of support could effectively alleviate their actual difficulties and psychological pressure encountered during training, which was an important external driving force to maintain their participation in multidomain interventions.

As for family support, family members were central in organizing intervention-related tasks, providing reminders, and accompanying patients during training sessions. The statements of the following three patients demonstrate strong family support.

*My family fully supports me. Whenever training requires something, they get it ready immediately. I never worry about facing difficulties.* (P1)

*I have a bad memory. Thanks to my husband, who reminds me on time every day and helps me arrange the training books and cards.* (P6)

*At the beginning, I was afraid when I came to a strange environment. My husband worried that I was nervous when I visited the clinic alone for training. He would accompany me every time. He was afraid that I could not understand the rules of the task game, and would ask the nurse to send him a copy each time. When he learned to teach me well, I would be willing to train and stick to it.* (P20)

As for peer assistance, it would provide patients with experiential knowledge, emotional encouragement, and a shared sense of belonging. Just like the two patients below said.

*Chen, who’s been training with me for half a year longer, often shares his experience with me. When I get stuck, he’s always there to guide me. Having him around keeps me motivated during my intervention.* (P7)

*We now have a mutual support group comprised of patients like myself. We regularly share training experiences and encourage one another whenever someone feels like giving up.* (P13)

Professional supervision was also frequently mentioned, as it was believed to influence participants’ engagement with the intervention. Participants indicated that medical staffs provided structured guidance, reminders, and a professional attitude, which supported their regular participation.

*The nurse sent us tasks every week with a special text message at the same time. I was embarrassed to be lazy.* (P5)

*You (nurses) are so strict, so professional, and have such a warm attitude. Well, I’ll stick with it then.* (P9)

*Every time I was in the clinic, the director would read my cognitive diary and guide the contents recorded in the diary. Considering that I had to hand in my homework every week, I would carefully record it.* (P11)

#### 3.2.4 Subtheme 4: high time preference during interventions

For patients with MCI, high time preference was defined as a tendency to prioritize immediate subjective experiences and short-term outcomes over long-term benefits during multidomain interventions. Participants frequently reported impatience with interventions requiring sustained investment of time and effort, resulting in emotional exhaustion, reduced motivation, and inconsistent adherence in most cases.

Some participants indicated that they might consider giving up or discontinuing the intervention if they did not experience any significant improvement or change in their cognitive abilities in the short term.

*I have been training for almost a month, and I do not feel that my memory is much better. I really do not want to go.* (P4)

*After each intervention, I did not feel any change, so I would be lazy occasionally and not come to training or do it at home.* (P9)

*The training teacher said that it would take at least 20 h to get some effect, but I wanted to see the change quickly…… If I did not make progress that day, I’d be worried at home and would not want to practice the next day.* (P15)

Some patients described that it was difficult to maintain their participation if they felt that interventions required a long-term commitment.

*Although I have started to participate in the intervention (multidomain intervention), I really cannot work hard on the thought of having to do it well for several months. Sometimes I simply miss several training sessions.* (P10)

#### 3.2.5 Subtheme 5: impact of frailty and comorbidities

Some MCI patients experience frailty and multiple comorbidities, making them prone to fatigue and physical discomfort during interventions and unable to sustain prolonged training sessions. Hearing and vision impairments limit their access to diverse training modules. Hearing and visual impairments limit their access to various training modules. Furthermore, the combination of multiple interventions increases patient fatigue.

One participant explained, *In addition to memory, I have obviously felt that my physical fitness has also declined in all aspects over the past two years. My legs hurt badly after walking a few steps. It took me a long time to go to the hospital for training. I really have no energy. Let us take care of my body first.* (P12)

Sensory impairment further limited participants’ engagement with certain interventions.

*My eyesight is really bad, I am easily tired from watching, and my hands are shaking. Now my physical condition is poor. I cannot persist in training every week. It’s pointless to go intermittently, so I have to ignore it first.* (P3)

Similarly, another participant reported that she had difficulty completing computer-based tasks due to dizziness and limited physical strength.

*The doctors and nurses said that my memory can only be improved if I persist in the intervention, but I cannot keep up with my physical strength when I’m old, especially when I sit up and look at the computer for a while, I feel dizzy, and I can only do half of the computer cognitive training each time.* (P16)

For individuals with limited physical strength, so many intervention programs in a multidomain intervention may leave them feeling exhausted.

*In the project, there are not only brain training tasks, but also sports, diet, and so on. I am thin and have limited energy, and I am prone to fatigue with more content.* (P20)

## 4 Discussion

This qualitative study investigated the authentic experiences and training journeys of MCI patients undergoing multidomain interventions. Eventually, this study identified two themes and eleven subthemes through a deductive-inductive approach.

To be specific, at the individual level, individual disease cognition plays a “double-edged sword” effect in behavior initiation. On the one hand, when patients perceived “abnormal aging” signals such as memory loss and declining learning, high-level health threats would urge them to understand and participate in intervention actively. It was the key to behavior initiation, as reported by Jiao et al. (28) and Sun et al. (29). The initiation of health behavior change is a transformation process from one behavior to another. In the MTM, an individual’s perception of health threats can activate the motivation for behavior change. As proposed by Gopalakrishnan et al. (30), individuals, when perceiving their health at risk, would actively take actions to reduce the risk. Consistent with Lu et al. (31), our study also noticed that some patients had a cognitive bias of attributing disease symptoms to “normal aging,” resulting in the lack of intervention needs, serving as one of the obstacles to behavior initiation. It is suggested that in clinical rehabilitation and nursing practice, attention should be paid to patients’ perception of disease threat (32), or it can be used as an important window to promote the initiation of multidomain interventions in patients with MCI. Additionally, for individuals with cognitive biases, it is important to implement diverse formats such as vivid case studies and educational videos, thereby enabling a clear distinction between normal aging and MCI by related patients, and effectively correcting misconceptions eventually. Our findings also revealed that some patients underestimated or denied their cognitive difficulties. Therefore, in addition to cognitive biases, some MCI patients, given their suffering from anosognosia, are unable to perceive the threat of the disease. Cognitive anosognosia is a disorder stemming from an underlying neurological dysfunction, rather than a disease-related cognitive bias, manifesting as impaired insight (33–35). Strategies based on education or awareness enhancement are only feasible if patients can self-recognize cognitive decline. However, these strategies may be ineffective or even unsuitable for subjects with cognitive anosognosia and are unlikely to translate into motivation for patient participation. Theoretically, this finding expands the application of perceived barriers and perceived benefits in participatory dialogue using the MTM. It also highlights a key boundary condition, namely, the assumption of sufficient self-awareness. In such cases, instead of depending solely on patient self-determination, the intervention may require greater reliance on external support, such as clinician advice or a systematic referral pathway.

Furthermore, patients’ decisions to initiate multidomain interventions may also be compromised by the structural barriers at the physical environment level. In our study, the identified barriers to accessing or utilizing interventions and the cost–benefit trade-offs revealed systemic deficiencies in information delivery, access to service resources, and health financing. When initiating such interventions, many patients with MCI may have limited access to information and resources related to the intervention, resulting in information asymmetry. It may be feasible by integrating cognitive health service recommendation mechanisms into community-based screening and hospital-level cognitive diagnosis, treatment, and rehabilitation processes, combined with proactive guidance and recommendations from healthcare professionals. It may eventually be conducive to the bridging of the information gap in service pathways (36). Beyond the insufficient provision of information on the supply side, information asymmetry is also embodied in the significant impact of differences in health literacy on patients’ ability to obtain, interpret, and evaluate cognitive health information and services (37). Patients with low health literacy may also find it difficult to assess their relevance and cost-effectiveness, in addition to struggling to identify available multidomain interventions, which may further erode their trust in healthcare institutions and systems (38). Owing to the shortage of health service resources and high transportation costs at the grass-roots level, there would be an exacerbated plight of “inaccessible” and “unaffordable” resources, as well as hindered delayed start of behavior (39). A study in Shanghai has confirmed that the availability of external resources was critical for initiating health behavior (31). The cost–benefit calculations of patients are adaptive decisions made in the context of limited insurance coverage, uneven service distribution, and out-of-pocket expenses. When intervention-related resources are unavailable (40) or participation is perceived as time- and cost-consuming (41), MCI patients will make a balance between participation and non-participation, resulting in a potential weakening in their participation motivation. At the policy level, there is a need for clearer institutional support and the development of standardized clinical guidelines to systematically evaluate the effectiveness and feasibility of multidomain interventions, which may help reduce uncertainty among patients and caregivers about their value (42). The problems of insufficient access to transportation and resources can be alleviated by expanding community and home-based intervention models, which are especially important for the elderly with mobility difficulties or those who live far from tertiary hospitals (43–45). Given the limited availability of skilled personnel, funding, and infrastructure, a complete integration of multidomain interventions into the primary care setting may not be immediately feasible as it necessitates significant financial and human resources (46, 47). In resource-scarce environments, it may be challenging to fully integrate multidomain interventions into primary healthcare systems immediately. In such cases, modular or trial-based intervention models may be a pragmatic alternative. These strategies may enable patients to experience potential benefits with minimal initial financial investment, thereby increasing their willingness to participate and gradually enhancing their understanding of the intervention’s value. Indeed, China’s National Basic Public Health Service Plan, launched in 2009, provides free public health services to the elderly. However, there is still a lack of programs specifically for cognitive rehabilitation (48). Currently, these interventions still require patients and their families to pay out of pocket. It is of great significance of expanding medical insurance coverage and incorporating cognitive rehabilitation-related services (e.g., multidomain interventions) into the public healthcare system at the policy level. It may facilitate the improvement of equitable access to multidomain intervention services for MCI patients and supporting their continued participation.

As described above, the initiation stage is primarily influenced by disease cognition and structural accessibility. Differently, the maintenance stage of multidomain interventions refers to a process of motivation internalization. Patients gradually transition from dependence on external supervision to developing self-regulation capabilities. Thus, behavior maintenance represents a different process, rather than a simple continuation of the initiation stage. Our study underscored a central role of positive training experiences in facilitating this internalization process. When the content of the training task fits the patient’s preferences and the needs are met, the patient can obtain a sense of responsibility and pleasure. This positive experience will become the internal driving force to maintain behavior, which is consistent with the research results of Bae et al. (49) and Szanton et al. (50). It can be interpreted that healthcare providers should enhance patient satisfaction and self-efficacy by tailoring training content to patient preferences, such as selecting tasks they enjoy, like craft activities. It is advisable to prioritize tasks that patients perceive as manageable yet still challenging, rather than exercises that are difficult to comprehend or exceed their capabilities. In addition, according to the interview results, social support such as family daily reminders, professional supervision of doctors and nurses, and peer experience sharing are the promoting factors of behavior maintenance, which is consistent with the research results of Chen et al. (51). A study (52) found that MCI patients often have strong emotional reactions such as anxiety and tension due to challenges such as cognitive impairment, dysfunction and social isolation. Their ability to recognize negative emotions is reduced, and they are more vulnerable to positive emotions (53, 54). Patients need comfort, and a strong external support system can effectively alleviate their psychological stress and negative emotions, and promote their continued engagement in interventions. Medical staff should extend their roles appropriately, focusing on building strong relationships with patients using various communication skills (55). At the same time, regularly monitor patients and provide them with feedback. It may improve trust, enhance the training experience, and reduce interruptions in the intervention. Family caregivers are an easily accessible source of external support that are closely integrated into the living environment of patients. Family caregivers can act as low-intensity support providers within the external support system. They can be integrated into patients’ daily life to offer daily reminders, companionship, and emotional encouragement to promote patient engagement (56, 57). It has been proposed that patients can receive peer support by participating in social activities (58). Therefore, MCI patients are organized to participate in group activities, share experiences through patient mutual assistance, encourage each other, enrich patients’ social support, and consolidate the motivation of patients’ behavior maintenance. Critically, external support has both advantages and disadvantages. To be specific, appropriate support can facilitate the maintenance of interventions. In contrast, excessive family supervision may increase the emotional burden on patients, foster dependency, and limit their opportunities to develop independent self-management skills. Meanwhile, peer support is inherently a relational and voluntary process. Its effectiveness decreases over time if group cohesion weakens, participation fluctuates, or peer networks disintegrate. It is worth noting that our study only focused on married and widowed individuals. Socially isolated patients or those without spouses or children face more challenges and may only have support from healthcare professionals or social workers, which is often time-limited and resource-dependent. This part of patients may struggle to maintain their behavior independently once formal supervision ends. To maintain multidomain interventions, it is necessary to help MCI patients gradually transition from the role of “patient” to the role of active self-manager, and to strike a balance between external support and autonomy (59).

As evidenced by our study, MCI patients can effectively promote behavior maintenance through appropriate self-regulation, while high time preference and physical function decline may exacerbate the risk of behavior interruption. MCI patients exhibited individual differences of self-regulation ability in behavioral practice. However, it has been documented previously that patients with a high cultural level and serious attitude had stronger adherence to the intervention (60). Thus, healthcare providers should design and deliver targeted self-management guidance for patients. It may involve instructing them on how to address temporary challenges encountered during training, consolidating successful experiences, and identifying signs of physical or cognitive fatigue. Such guidance may enable providers to promptly adjust intervention plans based on patient feedback, enhance patients’ self-efficacy, prevent premature abandonment of interventions following setbacks, and alleviate the burden on patients. At the same time, multidomain interventions require long-term adherence of MCI patients to achieve better results. FINGER and similar studies have shown that cognitive benefits in patients with MCI usually require interventions that last for several months or longer (4, 9, 61). Despite the recognized value of long-term adherence to intervention, some patients still face two challenges: the burden brought by high-frequency or long-term training goals, and the difficulty of resisting the “temptation of short-term escape,” as has been raised by Sugimoto et al. (12). Additionally, one participant in this study reported being told by a trainer that they would see the benefits after at least 20 h of training. Trainers who seek to encourage patient participation may inadvertently foster unrealistic expectations about the pace of cognitive change. In clinical settings, there is a mismatch between the typically provided intervention doses and those demonstrated to be effective in research trials. To lower participation barriers or improve adherence, simplifying intervention intensity or shortening duration is a frequent clinical implementation. The patient’s high time preference is a result of the intervention implementation failing to effectively “bridge” the gap between evidence-based dosage and real-world execution. In the design and implementation of interventions, it is crucial to provide patients with clear, staged, and achievable short-term and long-term goals. Moreover, the negative impact of delayed rewards should be mitigated by providing patients with tangible short-term process feedback (62). Improvements in cognitive function are typically progressive. Patients can be shown their progress through alternative, more flexible and proactive assessment feedback (e.g., comparisons of attention, physical function, or game performance). For patients with time constraints, flexible multidomain interventions can be designed by the medical staff to provide them with remote home training and allow them to accumulate training time according to their individual conditions. Noticeably, due to age-related lack of technological experience and cognitive impairments, it is not an easy task for the elderly with MCI using digital health technologies, which may hinder their ability to learn and use digital skills (63). In remote home training, the training process and platform interface can be simplified to improve usability for older adults with lower digital literacy, with assistance and guidance provided by medical staffs or family caregivers (64, 65). While we recommend guiding individuals and their families in learning to use digital training technologies, this assumes the presence of a caregiver or support person, which is not always the case for all patients. Therefore, the design of multidomain interventions should be integrated into daily life, and some computer-based tasks should have alternative intervention methods, such as traditional cognitive training, to ensure that those who cannot use digital platforms can still get access to these services (66). Additionally, frailty and comorbidities are underestimated but important factors in intervention maintenance. In real-world clinical settings, frailty often coexists with cognitive impairment and is the norm rather than the exception (67, 68). Katsurasako et al. (69) believed that patients would experience physiological load and fatigue due to physical function decline, producing negative impact on their participation and maintenance of multidomain interventions. Therefore, it is necessary to adopt appropriate adjustment for multidomain intervention strategies by healthcare providers, combined with dynamic modification of the difficulty of patients’ training tasks, reduction of reliance on limb movement and visual focus, implementation of dynamic monitoring during interventions to ensure patient safety, and potential involvement of family caregivers as “compensatory support” to assist patients in operating training equipment, thereby ensuring intervention continuity.

In terms of theoretical contributions, this study advances the development of the MTM by extending its application to older adults with MCI and complex multidomain intervention scenarios. By systematically mapping participants’ experiences onto the MTM framework, this study refines the explanation of key MTM structures in multidomain interventions for MCI patients, enriching the connotation of MTM. Simultaneously, this study demonstrates the applicability of MTM in analysing influencing factors of multidomain interventions for MCI patients, expanding the theoretical boundaries of MTM. This study provides a basis for future implementation of MTM-based multidomain interventions for MCI patients and offers valuable experience for the further development of MTM applications.

### 4.1 Limitations

In this study, our findings described the facilitators and obstacles to initiating and maintaining multidomain interventions in healthcare institutions and communities in urban areas. However, our samples were drawn solely from Nanjing, Jiangsu Province, China, implying that these findings may not be generalizable to all MCI populations. Future studies should be conducted based on the expansion of the geographical scope and the increase in the sample size. Furthermore, in our study, respondents with higher motivation, stronger reflective abilities, or more active participation in healthcare services would be more willing to share their experiences, potentially leading to an overrepresentation of supportive views on intervention participation or self-management, which might be a potential cause of selection bias. Simultaneously, patients might have social expectations when facing researcher questions, and patients’ willingness to participate in or adhere to interventions would be exaggerated owing to the use of self-reported interview data. Moreover, the retrospective nature of qualitative self-reported data relied on respondents’ recollections and interpretations of past experiences; patients might struggle to accurately recall or clearly articulate factors influencing their decision-making processes, and the reported motivations and obstacles were reconstructed narratives rather than objective descriptions of behaviors. Given that this study employed a cross-sectional qualitative research design, it was unable to fully capture the dynamic evolution of factors influencing intervention initiation and maintenance over time. This underscores the necessity of conducting future longitudinal studies. Building upon this study, we will employ a mixed-methods design, combining quantitative data to validate qualitative conclusions and conduct further in-depth research to track long-term behavioral changes in patients.

## 5 Conclusion

This study conducted semi-structured interviews with 20 patients with MCI. Based on the MTM and using a deductive-inductive approach, this study explored the influencing factors of the initiation and maintenance of multidomain interventions. This qualitative study discussed the feasibility of various suggestions to promote the initiation and maintenance of intervention, and provided the basis for medical staff to improve the implementation strategy of personalized multidomain interventions. In addition, we should promote the establishment of a closed-loop management system that spans from stimulating willingness to participate to sustaining intervention behaviors. This approach will enhance patient engagement and adherence to intervention measures, thereby facilitating the implementation of practical outcomes.

## Data Availability

The original contributions presented in the study are included in the article/Supplementary material, further inquiries can be directed to the corresponding author.
