# Supplementary material for: Influencing factors of initiation and maintenance of multidomain interventions in patients with mild cognitive impairment: a qualitative study
Source: Front Neurol. 2026 Jan 23;16:1734487. doi: 10.3389/fneur.2025.1734487 (PMC12875972; doi:10.3389/fneur.2025.1734487)
Supplement: Supplementary file 1 [file Supplementary_file_1.docx]

**Supplementary** **File 1. Semi-structured interview guide**

This semi-structured interview guide aims to explore the factors influencing the initiation and maintenance of patients with mild cognitive impairment (MCI) in multidomain interventions. Guided by a multi-theory model (MTM) of health behavior change, the guide has been continuously refined through literature review and pilot testing.

Core interview questions are intentionally open-ended, encouraging participants to describe their experiences in their own words. Probing questions are used flexibly based on participants' answers and experiences, rather than being a fixed checklist.

**1. Perceived cognitive changes:**

Did you experience memory decline? Please describe your feelings and how these changes affect you.

Probes：

①In what situations are you most likely to notice problems with your memory or attention?

②Will these changes affect your activities such as traveling, reading, cooking, managing your finances, or social interactions?

③What do you think are the reasons behind these changes?

④How do you typically respond to or handle these changes?

**2. Factors influencing participation in multidomain interventions：**

①Have you participated in a multidomain intervention program?

a. For patients with participation experience:

·Could you elaborate on your specific involvement in multidomain interventions?

·How did you learn about them?

·What factors motivated or hindered your participation?

Probes：

Cognitive Training:

·How have you found your experiences with memory or thinking exercises?

·Are these exercises easy or difficult to perform?

·What types of cognitive training have you participated in?

Physical Activity:

·How do you feel about the exercise component (e.g., intensity, frequency, physical exertion)?

Dietary Intervention:

·Have you received any dietary recommendations?

·What were these recommendations?

·Were they easy to understand and follow?

Social Activities:

·Has interacting with others affected your motivation, confidence, or enjoyment of participation?

Monitoring and Management of Vascular and Metabolic Risk Factors:

·Do you monitor your health indicators such as blood pressure or blood glucose and provide feedback to healthcare providers?

·Have changes in your health indicators affected your level of engagement?

b.For patients without participation experience: Would you participate in multidomain interventions if you got the opportunity? What factors influence your decision-making?

Probes：

·Which aspects of multidomain interventions (e.g., exercise, cognitive training, dietary advice, social activities, monitoring and management of vascular and metabolic risk factors) interest you most?

·Do you feel hesitant or resistant about certain parts? Why?

·What concerns do you have about participating (e.g., time commitment, physical condition, comprehension ability, costs)?

**3. Challenges and coping strategies during intervention**

What challenges have you encountered during your participation in multidomain interventions? Could you share your difficulties and how you addressed them?

Probes：

①Have you found certain components of the intervention more challenging than others?

②Did you experience physical fatigue, emotional stress, or frustration during your participation?

**4. Continue or stop participating**

Are you still actively participating in multidomain interventions? What motivates you to continue or discontinue your participation? What do you consider to be the most beneficial aspect of the intervention process?

Probes：

After experiencing certain parts of the intervention, did your motivation change?

**5. Expectations and support requirements**

What are your expectations and requirements regarding the content and format of multidomain interventions? What kind of support and assistance do you seek for?

Probes：

①What kind of guidance or feedback do you need most?

②Would support from family or community affect your participation?
